# The Utility and Feasibility of Laparoscopic Surgery in Patients Diagnosed With Cervical Cystic Lesions

**DOI:** 10.7759/cureus.64309

**Published:** 2024-07-11

**Authors:** Yu Horibe, Tsukuru Kamoshida, Ruriko Takase, Sakie Kashiwazaki, Toshiyuki Kanno, Takashi Motohashi, Yoshika Akizawa, Akira Nakabayashi, Jun Kumakiri, Tsutomu Tabata

**Affiliations:** 1 Obstetrics and Gynecology, Tokyo Women's Medical University, Tokyo, JPN

**Keywords:** mda, minimal deviation adenocarcinoma, laparoscopic, gas, gastric-type mucinous carcinoma, legh, endocervical glandular hyperplasia

## Abstract

Introduction: This observation study aimed to differentiate between lobular endocervical glandular hyperplasia (LEGH) and gastric-type mucinous carcinoma (GAS) while evaluating the feasibility and efficacy of laparoscopic surgery in the preoperative diagnosis of cervical cystic lesions.

Method and Material: A retrospective study was conducted to evaluate the diagnostic process and laparoscopic surgical management of cervical cystic lesions suspected to be LEGH or GAS. Preoperatively and postoperatively, MRI, cytology, histology, tumor marker analysis, and surgical outcomes (blood loss during surgery, operative time) were assessed. Six individuals were selected based on magnetic resonance imaging (MRI) results indicating a preoperative suspicion of LEGH or GAS. These patients underwent laparoscopic surgical treatment without indications of malignancy based on preoperative histology or cytology.

Results: Initially, all individuals were suspected to have LEGH based on MRI findings. Postoperatively, two patients were diagnosed with LEGH, two with adenocarcinoma in situ (AIS) and minimal deviation adenocarcinoma (MDA), and two showed no notable findings on pathology (one diagnosed endometrioid carcinoma in endometrial tissue). Patients with malignancies exhibited longer surgical times and higher intraoperative blood loss. Preoperatively, no significant variation was observed in maximal lesion diameter between adenocarcinoma and LEGH. However, lesion diameter increased significantly over time in patients with GAS.

Conclusion: Laparoscopic surgery demonstrated feasibility and provided crucial diagnostic and therapeutic outcomes, with no postoperative recurrence observed in cases of malignancy, despite the challenges associated with preoperative differentiation. These findings underscore the potential of laparoscopic surgery in enhancing both diagnostic accuracy and therapeutic efficacy for cervical cystic lesions, offering promise for improved patient outcomes and management strategies in clinical practice.

## Introduction

Cervical cystic lesions can occur in various histological types. However, differentiating lobular endocervical glandular hyperplasia (LEGH) from mucinous carcinoma gastric type (GAS) is difficult and often presents clinical problems. LEGH is a relatively new disease concept, first described in 1999 as having a benign histology similar to minimal deviation adenocarcinoma (MDA) [1]. MDA, in contrast, is considered an ultrastructurally hyperdifferentiated subtype of GAS, a clinicopathological entity. The tumor is aggressive and has a poor prognosis [2]. Both are similar in that they test positive for HIK1083 and MUC6 immunohistochemically, producing abundant gastric mucin [3,4]. Until recently, no existing method was capable of accurately differentiating them before surgery. Additionally, the LEGH-GAS sequence has been proposed as a precancerous lesion of GAS that should be considered from the viewpoint of surgical treatment [3].

As the diagnosis and treatment of LEGH and GAS have not yet been established, patients should be aware that excessive surgical invasion or underdiagnosis may lead to a poorer prognosis. In this study, we report a review of the preoperative diagnoses of LEGH and GAS as well as the utility and feasibility of laparoscopic surgery in patients with preoperative suspicion of LEGH and GAS.

## Materials and methods

This study is an observational, cross-sectional study in a single institution. Inclusion criteria are patients with suspected LEGH or GAS on preoperative examination, who underwent surgical treatment at our hospital between November 2021 and June 2024. The exclusion criterion is laparotomy. The eligible number of patients was found to be six. We compared age, surgical technique, operative time, intraoperative blood loss, magnetic resonance imaging (MRI) readings, cyst diameter in the cervix on MRI, cyst aggravation over time, shrinkage rate and days, tumor markers on blood examination, and postoperative pathology results to determine whether the disease was diagnosed before surgery and the effectiveness of laparoscopic surgery. Statistical tests were not performed due to the small population size and examined as means and variances.

## Results

Patient background and pathological results are presented in Table 1.

**Table 1:** Patient characteristics TLH=Total laparoscopic hysterectomy, BS=Bilateral salpingectomy, RASH=Robotic assisted simple hysterectomy, ATEC-US=Atypical endometrial cells of undetermined significance, AGC= Atypical glandular cells, AIS=Adenocarcinoma in situ, LEGH= Lobular endocervical glandular hyperplasia, GAS=Mucinous carcinoma gastric type, ACG-FN= Atypical glandular cells, Favor neoplastic, NILM= Negative for intraepithelial lesion or malignancy, NA=Not available.

Case	Age	Surgery	Surgery time (min)	Bleed (mL)	Cytology	Histology	MRI	MRI Diameter (mm)	Change of diameter before surgery (%)	Length of day for change diameter	Blood marker	Pathological diagnosis
1	56	TLH+BS	163	2	ATEC-US	Atypical glands	Suspected of LEGH	35x26	58	94	Negative	No Malignancy
2	45	TLH+BS	112	1	Negative	Suspected of LEGH	Suspected of LEGH	29x27	93	36	Negative	LEGH
3	45	RASH	274	30	AGC	No malignancy	Suspected of LEGH	39x36	71	292	Negative	atypical LEGH (AIS)
4	51	TLH+BS	212	1	NILM	No malignancy	Suspected of LEGH	26x28	92	189	Negative	LEGH
5	49	TLH+BS	288	100	AGC	No malignancy	Suspected of LEGH	25x25	120	230	Negative	GAS
6	44	TLH+BS	303	22	Negative	Negative	Suspected of LEGH	27x16	NA	NA	Negative	Negative (endometrioid carcinoma in endometrium)

First, all patients were suspected of having LEGH on MRI. Because there were no obvious malignant findings on cytology or histology before surgery, all patients underwent laparoscopic surgery, and no other abdominal surgeries were performed during the observation period. Only two out of six patients displayed no significant findings on postoperative pathology (one patient found endometrioid carcinoma, grade 1 in the endometrium, which is a different site from cervical tissue): two had LEGH and two had adenocarcinoma in situ (AIS) and MDA. In patients with malignancy, cervical cytology of the cystic lesions showed a diagnosis of atypical glandular cells (AGC), and there was a trend toward increased intraoperative blood loss and longer operative duration. All patients tested negative for tumor markers.

**Figure 1:**
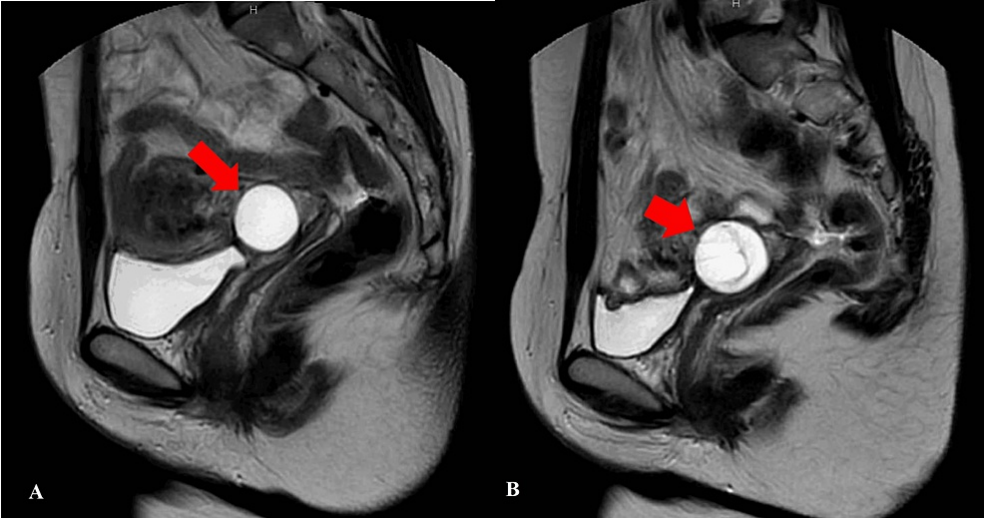
MRI images of the Case 5 patient A. MRI image at the first visit. No solid part is recognized in cervical cystic lesion; B. Second MRI just before surgery. An increase of 120% in diameter was found at 230 days in the mucinous carcinoma gastric type (GAS) case. The second MRI scan performed immediately before surgery showed no evident areas of enhancement or decreased diffusion. However, new septal structures were also observed.

No differences were observed in the maximum diameter between LEGH and adenocarcinomas in all cases. However, a comparison of the increase in the maximum diameter from the initial visit to the imaging examination immediately before surgery revealed that only patients with GAS (Case 5) exhibited a 120% increase in diameter at 230 days. Additionally, in these cases, the second MRI scan performed immediately before surgery showed no evident areas of enhancement or decreased diffusion. However, new septal structures were also observed (Figures 1A, 1B).

In patients 2 and 4, cervical histology indicated LEGH. Patients 2 and 4 were diagnosed with malignancy and were subsequently monitored without additional postoperative treatment such as chemotherapy and radiation therapy. No recurrence occurred following the initial surgery in all cases. In malignant cases, a follow-up period of two years and six months for patients with AIS (Case 3) and three years and five months for patients with MDA (Case 5) was suggested.

## Discussion

Our observation may identify challenges in distinguishing between LEGH and GAS while demonstrating the feasibility and effectiveness of laparoscopic surgery in diagnosing and managing cervical cystic lesions, with notable outcomes in cases of suspected malignancy. Cervical cystic lesions are a relatively new concept, with only a few reported cases. The mean age of onset for GAS is 42 years, with a five-year disease-free survival (DFS) rate of 74% for other adenocarcinoma types, contrasting with GAS’s poor prognosis at 38% [5]. This stark difference arises primarily from late-stage detection and a propensity for lesions to extend beyond cervical boundaries [6].

No standard protocol exists for treatment or diagnosis, and treatment decisions are currently made individually by patients and institutions. Based on our experience with a small patient cohort, we address the following three points: Indications for the surgical treatment of cervical cystic lesions, diagnosis of LEGH and GAS using preoperative examinations alone, and efficiency and feasibility of laparoscopic surgery for cervical cystic lesions.

For Indications for surgical treatment, according to Miyamoto et al. [7], surgical options such as observation, simple total hysterectomy, or radical hysterectomy depends on the clinical suspicion of a nabothian cyst, LEGH, or MDA; the presence of AGC or greater lesions on MRI or cervical cytology; or the detection of gastric-type mucin. Cervical conization is a viable diagnostic option. In our case, it aligns well with prior cytological diagnoses of AGC in both malignant cases. However, due to factors like advanced age, cervical atrophy, and difficulty in accessing the primary, we opted for a simple hysterectomy under a speculum for all premenopausal patients, confirming fertility status. The malignant transformation from LEGH to GAS is rare, occurring in approximately 1% [8]. Monitoring tumor diameter and cytological changes can aid early detection [9]. Comprehensive imaging evaluations, similar to those for other markers, are crucial. If the qualitative evaluation shows wall thickening of cystic lesions and the presence of solid components, aggressive treatment via total hysterectomy is warranted for both therapeutic and pathological exploration.

Next, diagnosis of LEGH and GAS using preoperative examinations alone, the results indicated that fully diagnosing LEGH or GAS before specimen retrieval was difficult. The low T2-weighted signal intensity of diffuse solids with indistinct boundaries within the LEGH or a solid component with restricted diffusion within the LEGH suggested the occurrence of GAS. In contrast, solid components are small and lack restricted diffusion, making complete differentiation difficult [5]. In addition, the lesion may have been more advanced intraoperatively than on MRI. The usefulness of positron emission tomography-computer tomography (PET-CT) is questionable because of the possibility of increased uptake due to inflammation and other factors [10].

On the other hand, a report shows GAS characteristics as follows; GAS generally shows at least focal atypia; it generally involves the entire cervical wall including the outer wall, in contrast to LEGH, which commonly affects only the inner wall; GAS usually presents as a poorly demarcated lesion, in contrast to LEGH which is usually well delimitated; GAS lacks lobular configuration, in contrast to LEGH which presents with a striking lobular architecture; and GAS can present desmoplasia and lymphovascular invasion [11].

Definitive diagnosis is based on the pathological confirmation of the presence of LEGH and GAS. However, the detection rate of cervical cytology and histology is low [12], and multiple examinations are required to improve diagnostic accuracy. Therefore, it is desirable to estimate the presence of LEGH or GAS by combining imaging, cytology, histology, blood sampling, ultrasonography, and other examinations.

Finally, we discuss the efficiency and feasibility of laparoscopic surgery for cervical cystic lesions. The prognosis of MDA is very poor, with a mean survival of approximately five years for patients with stage I, 38.1 months for patients with stage II, 22.8 months for patients with stage III, and 5.4 months for patients with stage IV [7]. In addition, no studies have compared laparoscopy and laparotomy for GAS and MDA in cervical lesions. Most reports by various authors have used abdominal surgery. However, the recurrence rate of MDA in advanced stages is high [13]. In this study, abdominal radical hysterectomy was selected when MDA or GAS was clearly confirmed by preoperative pathology and when there was evidence of disease extension beyond the cervix. However, if the lesion is not an initial or suspected lesion and our patients continue to have no evidence of disease (NED), laparoscopic hysterectomy is considered feasible. When performing laparoscopic surgery, techniques to prevent tumor spillage (intrauterine manipulation devices, fallopian tube sealing, and cervical sutures similar to those used in surgery for malignant cervical cancer) are necessary [14,15].

As a limitation, there is the inability to measure statistically significant differences due to small numbers of reports, and the inability to assess the long-term prognosis of malignant cases. Additionally, because no laparotomy cases were reported, we were unable to compare the prognosis, including the postoperative course, between laparotomy and laparoscopic surgery.

## Conclusions

Finally, MDA and GAS are associated with poor prognoses among malignant cervical lesions. Enhancing diagnostic accuracy involves integrating multiple examinations and contemplating laparoscopic surgery for early suspicious lesions. Future studies will incorporate additional cases to further investigate these conditions. In our observation, we found that laparoscopic surgery for cystic lesions of the cervix is feasible If the combined examinations are negative for malignancy and the indications for laparoscopic surgery are identified.

## References

[1] Nucci MR, Clement PB, Young RH (1999). Lobular endocervical glandular hyperplasia, not otherwise specified: a clinicopathologic analysis of thirteen cases of a distinctive pseudoneoplastic lesion and comparison with fourteen cases of adenoma malignum. Am J Surg Pathol.

[2] Gilks CB, Young RH, Aguirre P, DeLellis RA, Scully RE (1989). Adenoma malignum (minimal deviation adenocarcinoma) of the uterine cervix. A clinicopathological and immunohistochemical analysis of 26 cases. Am J Surg Pathol.

[3] Mikami Y, McCluggage WG (2013). Endocervical glandular lesions exhibiting gastric differentiation: an emerging spectrum of benign, premalignant, and malignant lesions. Adv Anat Pathol.

[4] Miyamoto T, Kobara H, Shiozawa T (2022). Biology and management of lobular endocervical glandular hyperplasia. J Obstet Gynaecol Res.

[5] Saida T, Sakata A, Tanaka YO (2019). Clinical and MRI characteristics of uterine cervical adenocarcinoma: its variants and mimics. Korean J Radiol.

[6] Park CM, Koh HM, Park S, Kang HS, Shim SS, Kim SY (2018). Gastric type mucinous endocervical adenocarcinoma of the uterine cervix: very rare and interesting case. Obstet Gynecol Sci.

[7] Ando H, Miyamoto T, Kashima H, Takatsu A, Ishii K, Fujinaga Y, Shiozawa T (2016). Usefulness of a management protocol for patients with cervical multicystic lesions: a retrospective analysis of 94 cases and the significance of GNAS mutation. J Obstet Gynaecol Res.

[8] Kobara H, Miyamoto T, Ando H (2020). Limited frequency of malignant change in lobular endocervical glandular hyperplasia. Int J Gynecol Cancer.

[9] Kobara H, Miyamoto T, Otsuki T, Ohya A, Shiozawa T (2020). Worsening cytology and lesion enlargement are useful indicators for malignant transformation of lobular endocervical glandular hyperplasia during follow-up: a case report. Gynecol Oncol Rep.

[10] Funakoshi M, Nakai G, Yamada T, Ohmichi M, Yamamoto K, Osuga K (2023). Acute cervicitis resembling gastric-type mucinous adenocarcinoma that was definitively diagnosed by cervical conization: a case report. Radiol Case Rep.

[11] Molero A, Parra A, Blanco I, Ascensión A, Ortega P (2023). Lobular endocervical glandular hyperplasia, a mimicker and potential pitfall for HPV-independent well differentiated gastric-type endocervical adenocarcinoma: case report and literature review focusing on histology, immunophenotype, and molecular findings. SAGE Open Med Case Rep.

[12] Li G, Jiang W, Gui S, Xu C (2010). Minimal deviation adenocarcinoma of the uterine cervix. Int J Gynaecol Obstet.

[13] Guo F, Hu Y, Xu X, Li R, Ru T, Wang J, Zhou H (2013). Diagnostic challenges in minimal deviation adenocarcinoma of the uterine cervix: a report of two cases and review of the literature. Mol Clin Oncol.

[14] Mun J, Park SJ, Yim GW, Chang SJ, Kim H (2021). Solution to prevent tumor spillage in minimally invasive radical hysterectomy using the endoscopic stapler for treating early-stage cervical cancer: surgical technique with video. J Gynecol Obstet Hum Reprod.

[15] Boyraz G, Karalok A, Basaran D, Turan T (2019). Vaginal closure with EndoGIA to prevent tumor spillage in laparoscopic radical hysterectomy for cervical cancer. J Minim Invasive Gynecol.

